# The New Year

**Published:** 1892-01

**Authors:** 


### HALL'S JOURNAL OF HEALTH

TRUTH DEMANDS NO SACRIFICE; ERROR CAN MAKE NONE.

Vol. 39. JANUARY, 1892. No. 1.

### THE NEW YEAR.

Once more we greet our friends and patrons with the compliments of the New Year.

With some we are brought face to face for the first time; others who received our greeting a year ago, have passed to the higher life, silvered o'er with the mellow ripeness of days well spent, and crowned with blessings; whilst many, very many old friends of the past, return our greetings with the same cordiality and good fellowship that have marked our intercourse in a period of years.

To one and all we extend our best wishes for their good health, happiness and long life.

